# Platelet factor 4-derived C15 peptide broadly inhibits enteroviruses by disrupting viral attachment

**DOI:** 10.1128/jvi.01859-24

**Published:** 2025-01-08

**Authors:** Shuai Lv, Congyi Li, Zhichao Pei, Ziwei Hu, Yining Du, Baisong Zheng, Wenyan Zhang

**Affiliations:** 1 Department of Infectious Diseases, Center of Infectious Diseases and Pathogen Biology, Institute of Virology and AIDS Research, Key Laboratory of Organ Regeneration and Transplantation of The Ministry of Education, The First Hospital of Jilin University 664674 , Changchun, Jilin, China; University of North Carolina at Chapel Hill, Chapel Hill, North Carolina, USA

**Keywords:** PF4, peptide C15, enterovirus, broad-spectrum, inhibitor

## Abstract

Platelet factor 4 (PF4) has been shown to regulate several viral infections. Our previous study demonstrated that PF4 inhibits the entry of enterovirus A 71 (EV71) and coxsackievirus A16 (CA16), which cause hand, foot, and mouth disease (HFMD). In this study, we report that PF4 also inhibits the circulating HFMD pathogen coxsackievirus A6 (CA6) and the re-emerging enterovirus D68 (EVD68). A 15-amino acid peptide, C15, at the C-terminus of PF4 confers anti-viral activity against multiple enteroviruses (EVs) besides CA6 and EVD68, including EV71 and CA16. Mechanistic studies revealed that wild-type C15 with a net-positive charge (+3), but not its mutants C15M and C15A (both −1), specifically binds to the VP3 capsid protein of CA6 and EVD68, thereby disrupting their attachment to the host cell surface. In addition, VP3 of EVs contains a conserved domain (residues 155–170) crucial for binding to C15. An aspartic acid residue at position 156 imparts a net-negative charge to this domain, which, when substituted with a neutrally charged amino acid, reduces the binding affinity of VP3 for C15. Additionally, C15 protects neonatal mice from lethal challenge upon a CA6 infection. These results suggest that C15 is a promising broad-spectrum anti-viral candidate against multiple EVs.

**IMPORTANCE:**

EVs, which pose a significant public health threat, can be classified into 15 species, with EV-A, -B, -C, and -D infecting humans and causing a wide range of diseases, from mild illnesses, such as HFMD, to more severe conditions, such as acute flaccid paralysis. The emergence of new and alternative strains highlights the urgent need for broad-spectrum anti-viral agents. In this study, we identified that the C15 of PF4 exhibits potent anti-viral activity against multiple EVs by binding to their surface and blocking their entry into host cells. Furthermore, C15 provides significant protection in vivo. These findings highlight the potential of C15 as a broad-spectrum anti-viral candidate. Our study opens a new avenue for developing treatments to combat the diverse and evolving threats posed by EVs.

## INTRODUCTION

Human enteroviruses (EVs) belong to the genus *Enterovirus* within the Picornaviridae family. Of the 15 species classified under EVs, four (EV-A, -B, -C, and -D) infect humans and cause a variety of diseases, ranging from mild conditions such as hand, foot, and mouth disease (HFMD) and herpangina to severe illnesses such as meningitis, encephalitis, myocarditis, respiratory disease, and acute ﬂaccid paralysis (AFP) (1, 2). Among these, enterovirus A 71 (EV71) and coxsackievirus A16 (CA16) were the primary causes of HFMD outbreaks during the period from 2007 to 2012 (3). In recent years, the prevalence of coxsackievirus A6 (CA6) and CA10 has significantly increased, with CA6 emerging as the major pathogen responsible for HFMD outbreaks worldwide (4). Enterovirus D68 (EVD68) was identified in 1962 as a respiratory virus in children with pneumonia and bronchiolitis (2). In 2014, EVD68 re-emerged, causing a widespread outbreak of severe respiratory illness in North America, followed by a biennial pattern typical of certain EV types in the United States, South America, and Europe (5–8). Although an EV71 vaccine has been available in China since 2015, providing 80% and 90% protection against severe and mild HFMD, respectively (9), it does not protect against infections caused by other EV subtypes. The lack of multivalent protective vaccines and effective broad-spectrum anti-virals makes EVs a significant public health concern.

EVs undergo a typical viral life cycle of attachment, entry, uncoating, replication, translation, assembly, and release. Various cell surface receptors, such as human scavenger receptor class B member 2, P-selectin glycoprotein ligand-1, heparan sulfate glycosaminoglycan (HS), sialylated glycan, annexin II, vimentin, intercellular adhesion molecule 5/telencephalin, fibronectin, and prohibitin, are utilized by different EVs for attachment (10–15). The capsid proteins of EVs that bind to these receptors are critical for viral attachment and entry, making the disruption of these interactions an important target for anti-viral drugs. Soluble SCARB2 and monoclonal antibodies, such as JL2, A9, and D6, have emerged as promising treatments for EV71 infection (14, 16, 17). Additionally, the chemosynthetic EV71 polypeptide SP40, which carries a positive charge, has been shown to prevent viral attachment and effectively reduce EV71 infection (18). Other strategies, including HS analogs or small molecules targeting EV71 VP1, have been developed and validated mainly *in vitro* (19, 20). Given the emergence of various epidemic EV strains, there is a critical need to develop effective broad-spectrum anti-EV drugs for treating acute infections.

Platelet factor 4 (PF4), also known as CXC chemokine 4, is secreted by platelets (21). PF4 has been shown to regulate infections by various viruses, including human immunodeficiency virus type I (HIV-1), respiratory syncytial virus (RSV), H1N1 influenza, dengue virus, and Japanese encephalitis virus (22–26). Our recent study demonstrated that PF4 inhibits cellular entry of EV71 and CV16, thereby inhibiting their replication (27). Previous studies have highlighted several novel anti-viral peptides, such as BanLec, which inhibits HIV, hepatitis C virus, and influenza virus; theta-defensin retrocyclin 2, which targets influenza virus, Sindbis virus, and baculovirus; and P9R, which is effective against H1N1, H7N9, and coronaviruses (28, 29). Peptide drugs have also demonstrated clinical efficacy, with enfuvirtide and aprepitant being used in the treatment of HIV infection (30). However, there are currently no specific anti-viral drugs approved for EV infections, as numerous small-molecule drugs have failed in phase II and III clinical trials (31). Therefore, peptide drugs may represent a more effective alternative for combating EV infections.

In this study, we designed a 15-amino acid peptide, C15, derived from PF4. C15 demonstrated broad-spectrum anti-viral activity at nanomolar concentrations against multiple EVs, including CA6, EVD68, EV71, and CA16. We confirmed that C15 acts as an entry inhibitor by binding to the VP3 protein of EVs, thereby disrupting their interaction with cellular attachment receptors. Additionally, C15 effectively protected neonatal mice from lethal challenge with CA6, highlighting its potential as an effective therapeutic strategy for EV infections.

## RESULTS

### PF4 inhibits the replication of CA6 and EVD68

**Fig 1:**
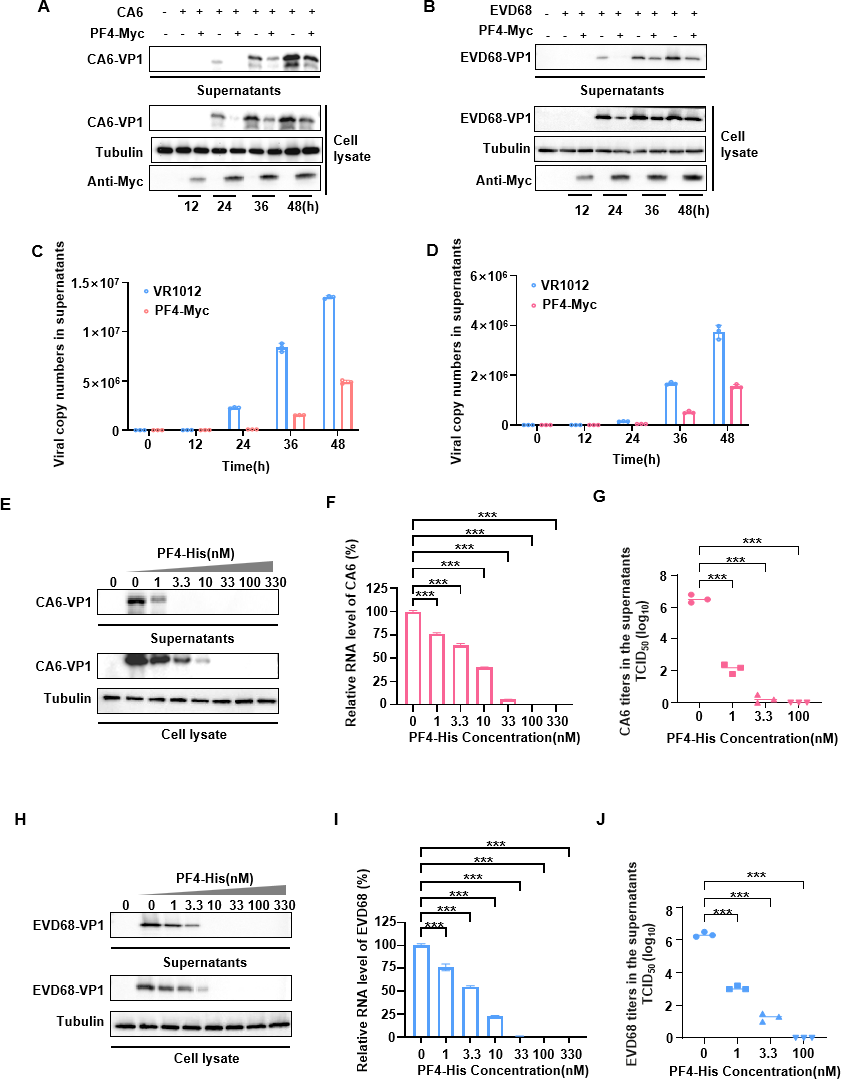
PF4 inhibits the replication of CA6 and EVD68. (**A and B**) HEK-293T cells transfected with cDNA-PF4-Myc or the VR1012 expression vector serving as a control. After 12 h, the cells were infected with CA6 (MOI = 0.5) or EVD68 (MOI = 1). At the indicated time points, viral loads in both the cells and the supernatant (concentrated 100-fold) were assessed using Western blot analysis with an anti-VP1 antibody, while tubulin was used as a loading control. (**C and D**) Viral loads in the supernatant were evaluated by measuring VP1 gene mRNA levels using reverse transcription quantitative PCR (RT-qPCR). (**E and H**) PF4-His proteins expressed in *Escherichia coli* were added to the cell supernatant at the indicated concentration, and the cells were simultaneously infected with CA6 or EVD68. Cell lysates and supernatants (concentrated 100-fold) were collected at 48 h. Western blot was performed to detect the viral VP1 protein in the cells and supernatant, with tubulin serving as a loading control. (**F and I**) Intracellular mRNA levels of VP1 were determined by RT-qPCR, using GAPDH as a control. (**G and J**) 50% tissue culture infective dose (TCID_50_) assays were performed using supernatants collected from infected rhabdomyosarcoma cells at 48 h, containing various concentrations of PF4-His. All data are derived from three independent experiments and are presented as mean ± SD. Statistical significance was determined using one-way analysis of variance. ***P < 0.001.

CA6 is the most prevalent causative agent of HFMD worldwide (4). EVD68 is a re-emerging pathogen of public health concern and has been associated with severe AFP (2, 32). To determine whether PF4 inhibits the replication of CA6 and EVD68, human embryonic kidney (HEK293T) cells were transfected with PF4 tagged with hemagglutinin (HA) or VR1012 (negative control) plasmids, then infected with CA6 or EVD68 at a multiplicity of infection (MOI) of 0.5 and 1.0, respectively. PF4 overexpression suppressed the replication of CA6 and EVD68 as is evident in VP1 protein levels of intracellular and in the culture supernatant over time, especially after 24 h post-infection (Fig. 1A and B). PF4 also decreased viral load of CA6 and EVD68 in the supernatant by detecting VP1 mRNA levels (Fig. 1C and D).

PF4 is a secreted protein. To further assess its anti-viral potential, PF4 proteins were expressed and purified from *Escherichia coli*. Increasing doses of purified PF4-His were added to the cells infected with CA6 or EVD68, as indicated. PF4 gradually decreased VP1 protein and mRNA levels of CA6 and EVD68 in both intracellular compartment and viral supernatants, as well as the viral titer in the supernatant in a dose-dependent manner (Fig. 1E through J), suggesting that exogenous PF4 can inhibit EV replication.

### C-terminus of PF4 is necessary for CA6 and EVD68 inhibition

**Fig 2:**
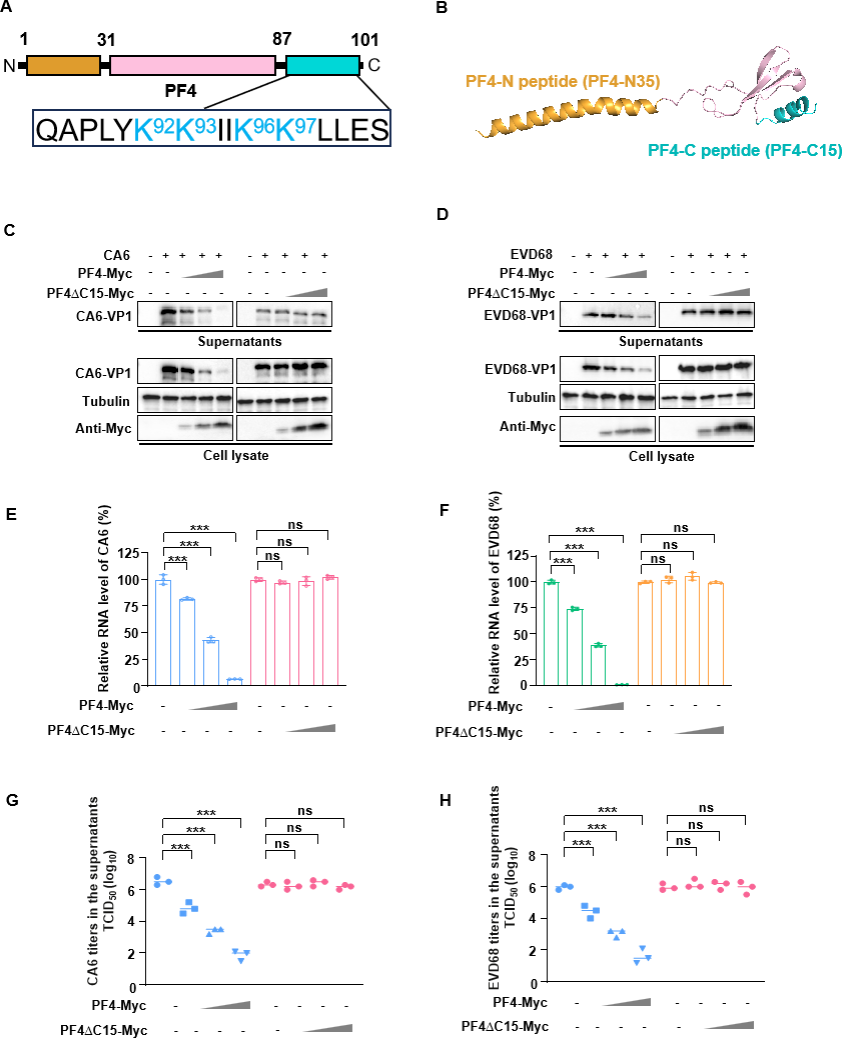
PF4 lacking 15 amino acids at the C-terminus loses its anti-viral activity. (**A**) Schematic representation of the PF4 gene. (**B**) Prediction of the three-dimensional (3D) structure of the PF4 protein using AlphaFold2. (**C and D**) HEK-293T cells were transfected with varying concentrations of cDNA-PF4-Myc, with the VR1012 expression vector as a control. Twelve hours later, the cells were infected with CA6 or EVD68. Western blot analysis was performed to detect the expression levels of various proteins using the corresponding antibodies. (**E and F**) RT-qPCR was used to measure the mRNA levels of VP1 of CA6 or EVD68 in cells transfected with different concentrations of cDNA-PF4-Myc. GAPDH was used as a normalization control. (**G and H**) TCID_50_ assays were performed using supernatants collected from infected rhabdomyosarcoma cells at 48 h, containing various concentrations of PF4-Myc. All data are derived from three independent experiments and are presented as mean ± SD. Statistical significance was determined using one-way analysis of variance. **P < 0.001. ns, no significance.

To identify which domain of PF4 is required for EV inhibition, we analyzed the sequence of PF4 and predicted its structural model using the AlphaFold 2 (32) program (Fig. 2A and B). The predicted structure divided the PF4 protein into three domains: the N-terminal signal peptide, the central region, and a 15-amino acid sequence at the C-terminus. Since the mechanisms of action of peptide drugs are often related to the polarity of their amino acids, it is noteworthy that the C-terminus of PF4 is highly polar. Based on the predictive structure, we constructed a PF4 mutant lacking the C-terminus (PF4-ΔC15) and examined its effect on EV replication inhibition. This mutant lost the ability to inhibit the replication of CA6 and EVD68 (Fig. 2C through H), even at the highest concentration compared to the relatively low dose of wild-type (WT) PF4 (PF4-WT).

**Fig 3:**
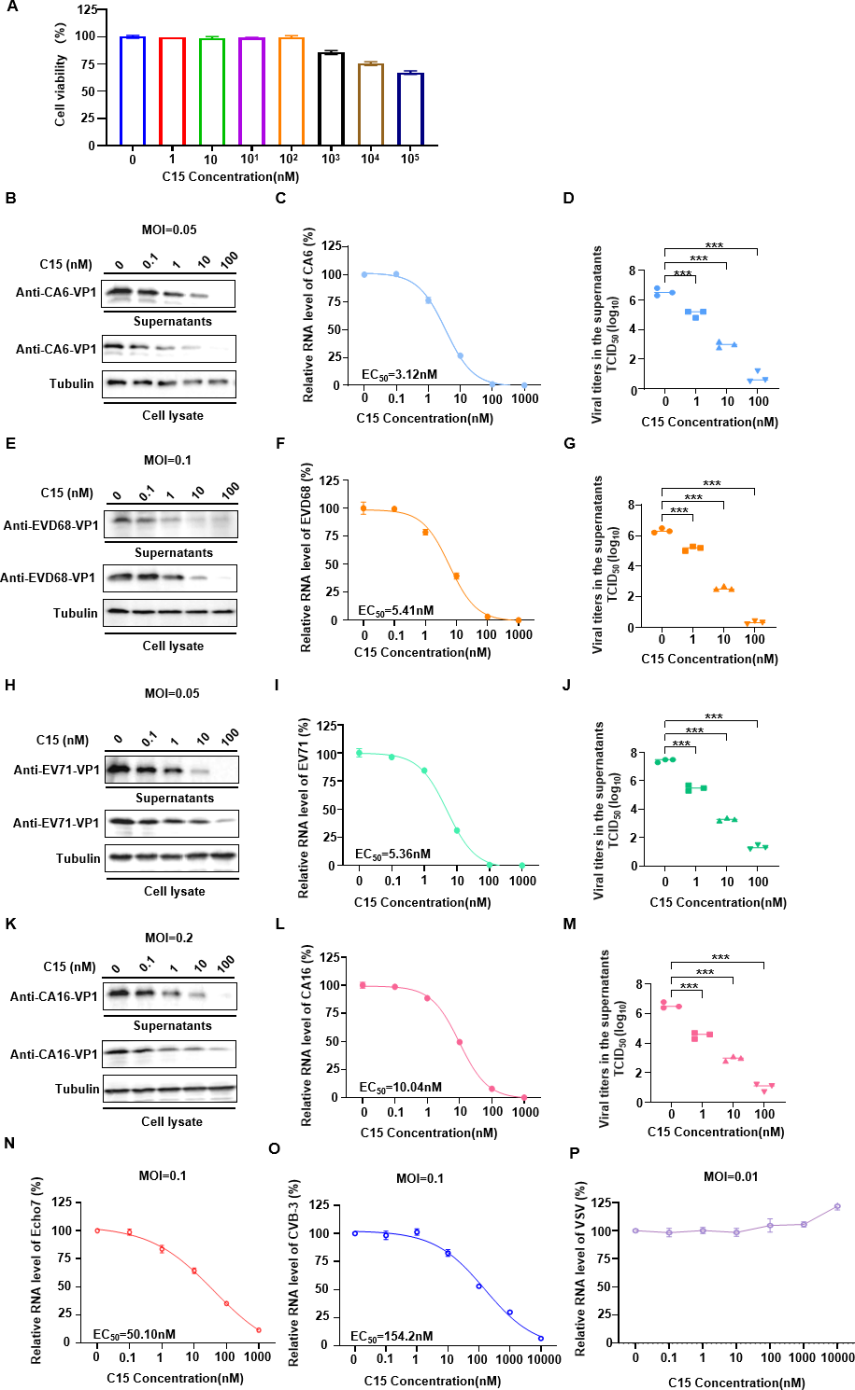
C15 inhibits the replication of multiple EVs. (**A**) Cell viability of rhabdomyosarcoma (RD) cells after 48 h treatment with different concentrations of C15 was assessed using the Cell Counting Kit-8 assay (*n* = 3). (**B, E, H, and K**) RD cells were infected with indicated enterovirus (at various MOIs) and treated with different concentrations of C15 for 48 h. Western blot analysis was conducted to detect the VP1 protein levels in cell lysates and supernatants (concentrated 100-fold), with tubulin used as a loading control. (**C, F, I, and L**) Intracellular mRNA levels of the *VP1* gene from different enterovirus strains were quantified by RT-qPCR, with GAPDH as a normalization control. All data are from three independent experiments and are presented as mean ± SD. The EC_50_ values were determined using GraphPad Prism version 9 with a variable slope (four parameters). (**D, G, J, and M**) TCID_50_ assays were performed using supernatants collected from infected RD cells at 48 h, containing various concentrations of C15. All data are from three independent experiments and are presented as mean ± SD. Statistical significance was assessed using one-way analysis of variance. ***P < 0.001. (**N, O, and P**) Intracellular mRNA levels of different enterovirus strains were quantified by RT-qPCR, with GAPDH as a normalization control. All data are from three independent experiments and are presented as mean ± SD. The EC_50_ values were determined using GraphPad Prism version 9 with a variable slope (four parameters).

To further confirm that the C-terminus of PF4 is required for EV inhibition, we synthesized a 15-amino acid peptide (C15) located at the C-terminus of PF4 and examined its cytotoxicity and inhibitory effect against multiple EVs. The 50% cytotoxic concentration value of C15 was >100 µM (Fig. 3A). Most importantly, C15 strongly suppressed the replication of several EVs, including CA6, EVD68, EV71, and CA16, as shown by reduced intracellular VP1 protein and mRNA levels, as well as lower viral titers in the supernatant (Fig. 3B through M). The EC_50_ values of C15 for CA6, EVD68, EV71, and CA16 were 3.12, 5.41, 5.36, and 10.04 nM, respectively. Simultaneously, we examined the effect of C15 on Echo7 and CVB3 replication, which are classified into enterovirus type B. The results showed that C15 also inhibited the replication of these two viruses, although its efficacy was not as strong as that observed with four abovementioned viruses (Fig. 3N and O). Furthermore, C15 was unable to inhibit the replication of vesicular stomatitis virus (Fig. 3P), suggesting that C15 may exhibit specificity for enterovirus replication. Collectively, these results demonstrate that C15 exhibits broad-spectrum inhibitory activity against multiple EVs.

### The inhibition of C15 on CA6 and EVD68 occurs at the entry stage

**Fig 4:**
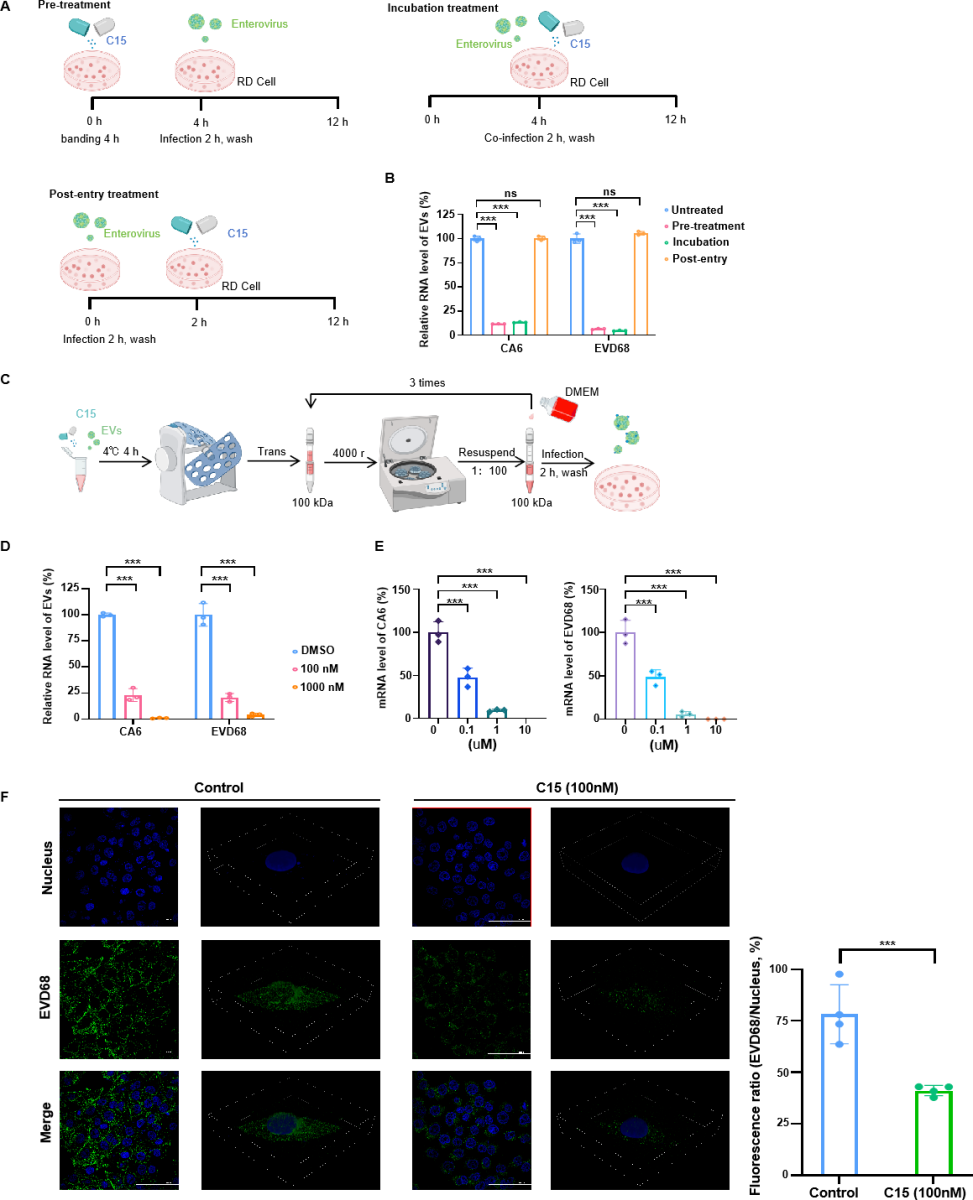
C15 inhibits viral entry into cells by affecting virus binding to cells. (**A and B**) A time-of-addition assay with C15 was performed. RD cells were treated with C15 at different stages: pretreatment, incubation treatment, or post-entry of CA6 (MOI = 0.05) and EVD68 (MOI = 0.1). The inhibitory effect was assessed by measuring intracellular vRNA concentrations at 12 h post-infection (*n* = 3). (**C and D**) CA6 and EVD68 were incubated with C15 in an Eppendorf (EP) tube for 4 h at 4°C. The mixture was then concentrated 1:100 and concentrated using a 100 kDa protein concentrator tube, with three exchanges to remove excess C15. The final virus solution was re-suspended in cell culture medium and used to infect RD cells. After 48 h, cells were collected and analyzed by RT-qPCR. (**E**) RD cells in six-well plates were infected with CA6 (MOI = 0.5) and EVD68 viruses containing C15 (100 and 1,000 nM) at 4°C for 1 h. The cells were then washed three times with cold phosphate-buffered saline, lysed, and vRNA levels were measured by RT-qPCR. (**F**) EVD68 virus bound to the surface of HeLa cells was detected by immunofluorescence. Multicellular (flat image) and single cell (stereoscopic 3D image) are shown. Bars of flat image are 100 μm, and bars for 3D image are as follows: length, 58 µm; width, 58 µm; height, 12 µm. The histogram shows the fluorescence ratio. All data are from three independent experiments and are presented as mean ± SD. Statistical significance was assessed using one-way analysis of variance. ***P < 0.001. ns, no significance.

To further investigate which stage of C15 functions on CA6 and EVD68 infections, we designed three assays as follows: (i) C15 pretreatment in which C15 was added to rhabdomyosarcoma (RD) cells for 4 h before EV attachment, (ii) incubation treatment in which C15 and EVs were incubated for 4 h *in vitro* before being added to RD cells, and (iii) post-entry treatment in which RD cells were infected with CA6 or EVD68 for 2 h before C15 was added (27) (Fig. 4A). The results showed that both C15 pretreatment and incubation of C15 and EVs significantly inhibited the replication of CA6 and EVD68. In contrast, post-entry treatment with C15 failed to inhibit viral replication (Fig. 4B). This suggests that C15 blocks viral entry by interacting with the receptor or viral proteins on the surface, thereby interfering with the entry of CA6 and EVD68 into the cells. Furthermore, when we pretreated the viruses with C15 for 48 h and then removed the excess C15 by centrifugation before infecting the cells, the C15 bound to the viruses and inhibited their replication (Fig. 4C and D). Furthermore, centrifugation did not disrupt the interaction of C15 and the viruses, indicating a strong interaction between C15 and the viral particles. Viral attachment experiments further demonstrated that C15 inhibited the ability of the virus to adsorb onto RD cells by binding to the virus (Fig. 4E). Similar observations were noted when the inhibition of EVD68 attachment by C15 was visualized using immunofluorescence in HeLa cells (Fig. 4F).

### PF4-ΔC15 failed to interact with EV VP3 protein

**Fig 5:**
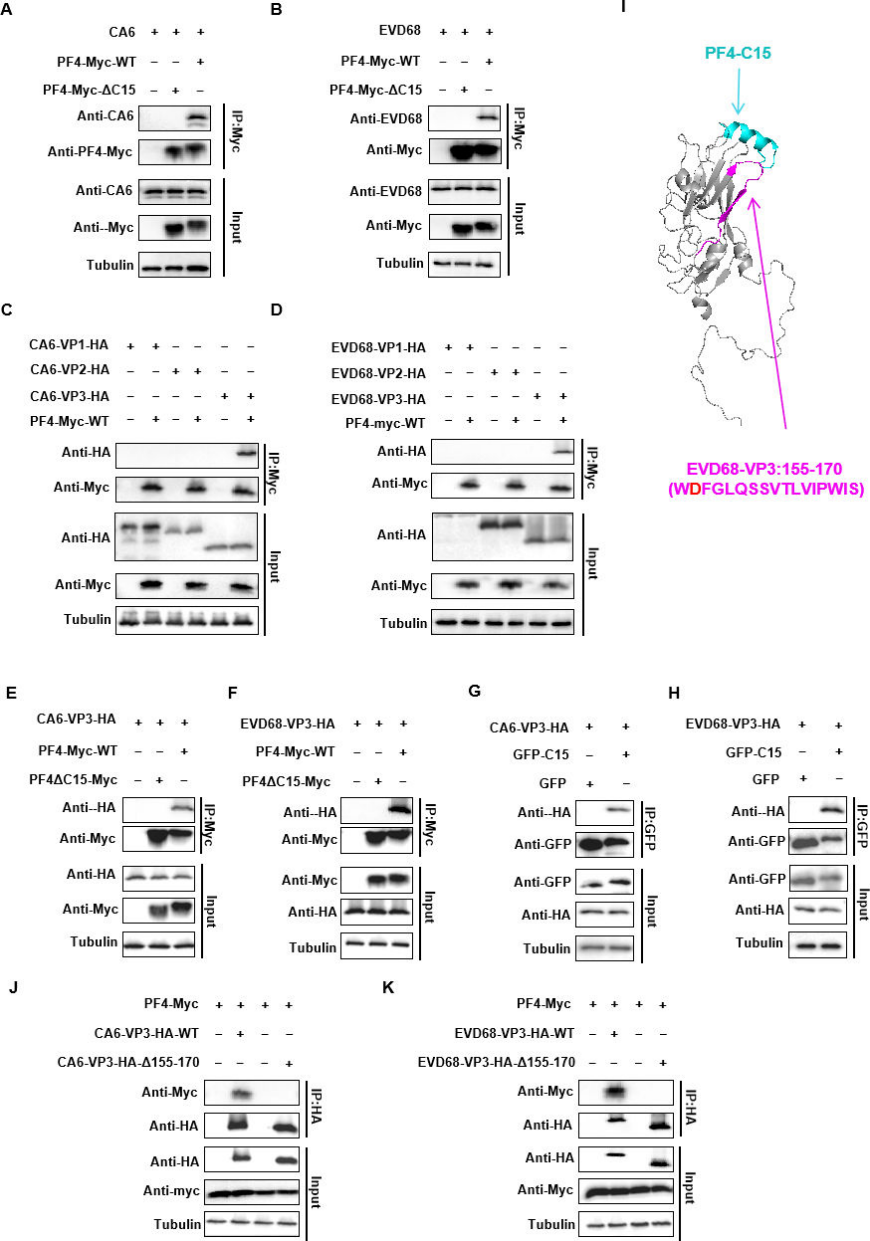
PF4 interacts with the VP3 proteins of CA6 and EVD68. (**A and B**) HEK293T cells were transfected with cDNA-PF4-Myc or PF4△C15-Myc. The cell lysates were incubated with protein G containing Myc antibody, then CA6 and EVD68 suspensions were added for 4 h, followed by co-IP analysis. VR1012 expression vector served as a control. (**C and D**) Co-IP analysis of the interaction between CA6/EVD68-VP1/2/3-HA and PF4-Myc in HEK293T cells transfected with the indicated plasmids. VR1012 expression vector was used as a control. (**E and F**) Co-IP analysis of the interaction between CA6/EVD68-VP3-HA and PF4-Myc or PF4ΔC15-Myc in HEK293T cells transfected with the indicated plasmids. VR1012 empty vector served as a control. (**G and H**)Co-IP analysis of the interaction between CA6/EVD68-VP3-HA and GFP-C15 in HEK293T cells transfected with the indicated plasmids. p-EGFP expression vector was used as a control. (**I**)Docking result showing the interaction between C15 and EVD68-VP3. Blue, C15; gray, EVD68-VP3; purple, EVD68-VP3 interaction region (155–170). (**J and K**) Co-IP analysis of the interaction between CA6/EVD68-VP3-HA (wild type or Δ155–170) and PF4-Myc in HEK293T cells transfected with the indicated plasmids. VR1012 expression vector was used as a control.

The previous experiments showed that C15 pretreatment affected viral attachment. To determine whether PF4 via C15 directly interacts with viral particles, we conjugated PF4-WT or PF4-ΔC15 with a Myc tag to protein G beads, incubated them with CA6 and EVD68 viral suspensions, and performed co-immunoprecipitation (co-IP). The results showed that only PF4-WT was able to pull down CA6 or EVD68 virions, whereas PF4-ΔC15 was not (Fig. 5A and B). To identify which viral proteins of CA6 or EVD68 are necessary for PF4 binding, we performed a co-IP of PF4-WT with the VP1, VP2, and VP3 capsid proteins on the surface of CA6 and EVD68. The results revealed that PF4 interacted with VP3 of both viruses, which is consistent with our previous findings with VP3 of EV71 and CA16 (Fig. 5C and D). As expected, PF4-ΔC15 did not pull down any of the VP3s (Fig. 5E and F). However, C15 with a green fluorescent protein (GFP) tag at the C-terminus (GFP-C15) pulled down VP3, whereas GFP alone did not (Fig. 5G and H). Next, we used the zDOCK website to model the docking of C15 with EVD68 VP3 and predicted their binding sites. The primary binding site on VP3 for C15 is located at amino acid residues 155–170, which is a conserved domain among EVD68, CA6, EV71, and CA16 (Fig. 5I). To test this prediction, we performed a co-IP with PF4 using VP3-WT or a VP3 mutant lacking the 155–170 (WDFGLQSSVTLVIPWIS) residues (VP3-Δ155–170). As expected, the VP3-Δ155–170 mutant was unable to pull down PF4, indicating that the absence of these residues disrupted the interaction (Fig. 5J and K). These results confirm that PF4 interacts with VP3 through its C-terminal peptide C15, with amino acid residues 155–170 of VP3 serving as the primary binding site for C15.

### C15 efficiently interacts with the conserved domain of EV VP3 proteins

**Fig 6:**
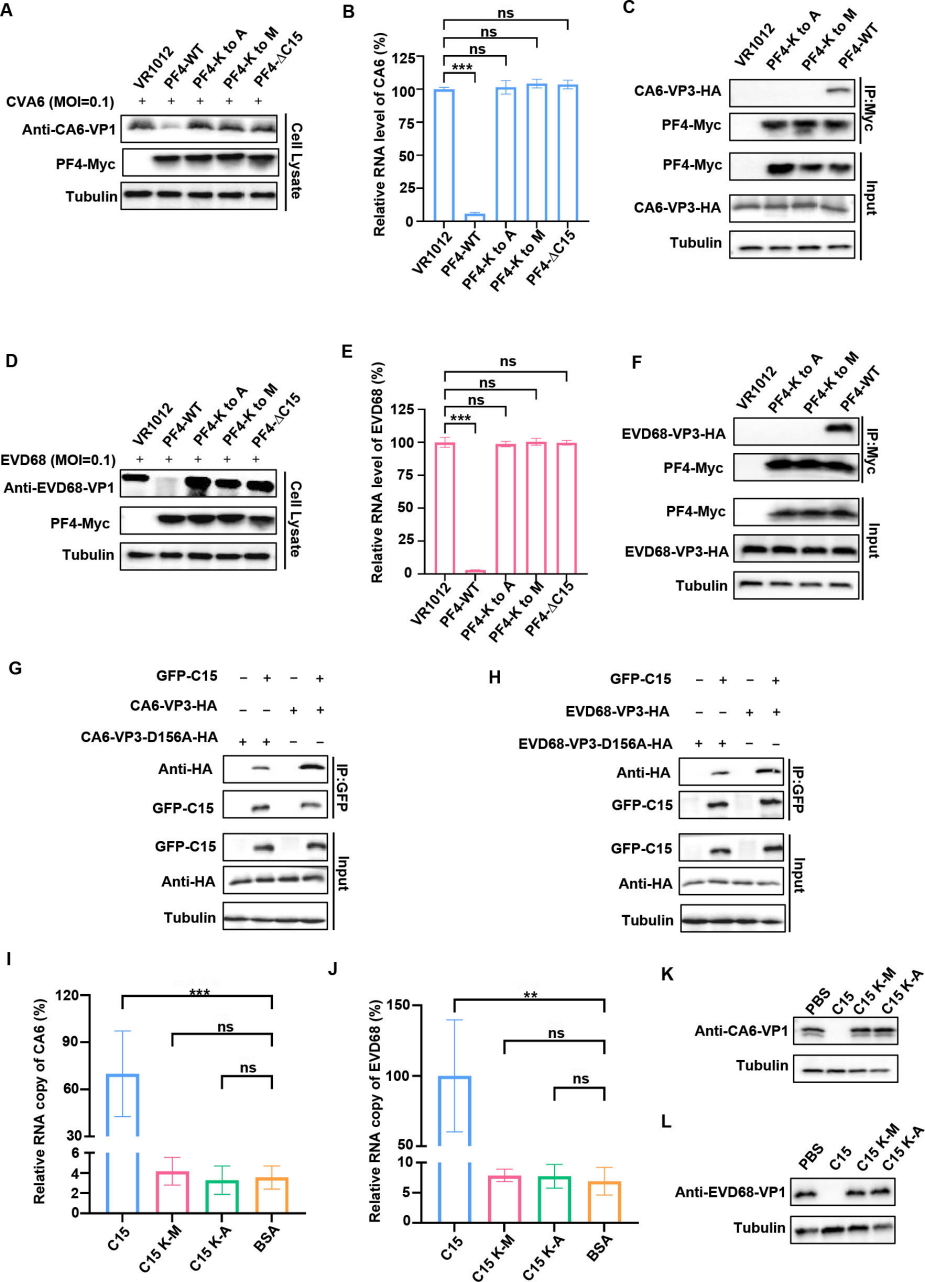
The interaction between C15 and the VP3 proteins of CA6 and EVD68 via a direct ionic interaction. (**A and D**) HEK-293T cells were transfected with cDNA-PF4-Myc or its mutants, with the VR1012 empty vector serving as a control. After 12 h, the cells were infected with CA6 (MOI = 0.5) and EVD68 (MOI = 1). Viral productions were assessed using Western blot analysis with an anti-VP1 antibody at 48 h, while tubulin was used as a loading control. (**B and E**) Viral mRNA levels in the cells were evaluated by quantifying VP1 mRNA levels using RT-qPCR. (**C and F**) Co-IP analysis of the interaction between CA6/EVD68-VP3-HA and PF4-Myc or its mutants in HEK293T cells transfected with the indicated plasmids. VR1012 expression carriers were used as a control. (**G and H**) Co-IP analysis was conducted to investigate the interaction between CA6/EVD68-VP3-HA or their D156A mutants and PF4-Myc in HEK293T cells transfected with the indicated plasmids. The VR1012 expression vector served as a control. (**I and J**) The binding ability of peptide C15 and its mutants to CA6 and EVD68 was assessed by ELISA and RT-qPCR analysis (*n* = 3). The relative RNA copy number of the virus binding to peptides was normalized to the binding of the virus to C15 and its mutants. (**K and L**) At a concentration of 100 nM, peptide C15 effectively inhibited the replication of CA6 and EVD68, whereas its mutant did not exhibit such inhibitory effects. Three independent experiments are presented as mean ± SD. Statistical significance was assessed using one-way analysis of variance. **P < 0.01, ***P < 0.001. ns, no significance.

EVs are negatively charged in physiological environments (33, 34). Previous studies showed that several small peptides bind to viruses, relying on the abundance of basic amino acids in their composition (29, 35). These basic amino acids contribute to a positive charge on the peptides. Using the InnovaGen website, we predicted that C15 carries a net-positive charge of +3, attributed to its four lysine (K) residues. Mutating these residues to neutrally charged amino acids, such as alanine (A) or methionine (M), results in a peptide with an overall charge of −1 (Table 1). To assess the role of amino acid charge in peptide-virus interactions and viral inhibition, we introduced mutations into the PF4 expression vector. The results showed that PF4-C15 mutants, with a K to A or a K to M change, and the PF4-ΔC15 mutant all lost their anti-viral activity (Fig. 6A, B, D and E) and were unable to pull down VP3 (Fig. 6C and F), indicating that the interaction of PF4-C15 with the virus is influenced by amino acid charge. The critical binding region of VP3, residues 155–170, has a net-negative charge of −1, primarily due to the aspartic acid (D) at position 156. To investigate the role of this negative charge in the interaction of VP3 with C15, we mutated the D at position 156 to an A in VP3 (VP3-D156A). Co-IP showed that the binding ability of this VP3-D156A mutant to GFP-C15 was significantly weaker than that of VP3-WT (Fig. 6G and H). Subsequently, the C15 peptide and its mutants (C15K-A, C15K-M) were immobilized on a microplate plate; the virions were captured by enzyme-linked immunosorbent (ELISA), and the viral RNA was quantified by reverse transcription quantitative PCR (RT-qPCR). The result showed that only the WT C15 peptide could bind to CA6 and EVD68 virions, whereas the mutant C15 peptides carrying an A or a K mutation failed to bind (Fig. 6I and J). Furthermore, these mutated peptides did not inhibit the replication of CA6 and EVD68, confirming the loss of their anti-viral activity (Fig. 6K and L). In summary, these findings suggest that PF4 binds to VP3 through an interaction between their net-positive and net-negative charges, respectively.

**TABLE 1:** Analysis of peptide sequences and their electric charges by PepCalc from InnovaGen

PF4-C15	Amino acid sequence	Electric charge
C15-WT	QAPLYKKIIKKLLES	+3
C15 K-M	QAPLYMMIIMMLLES	−1
C15 K-A	QAPLYAAIIAALLES	−1

### C15 treatment protects neonatal mice from CA6 lethal challenge

**Fig 7:**
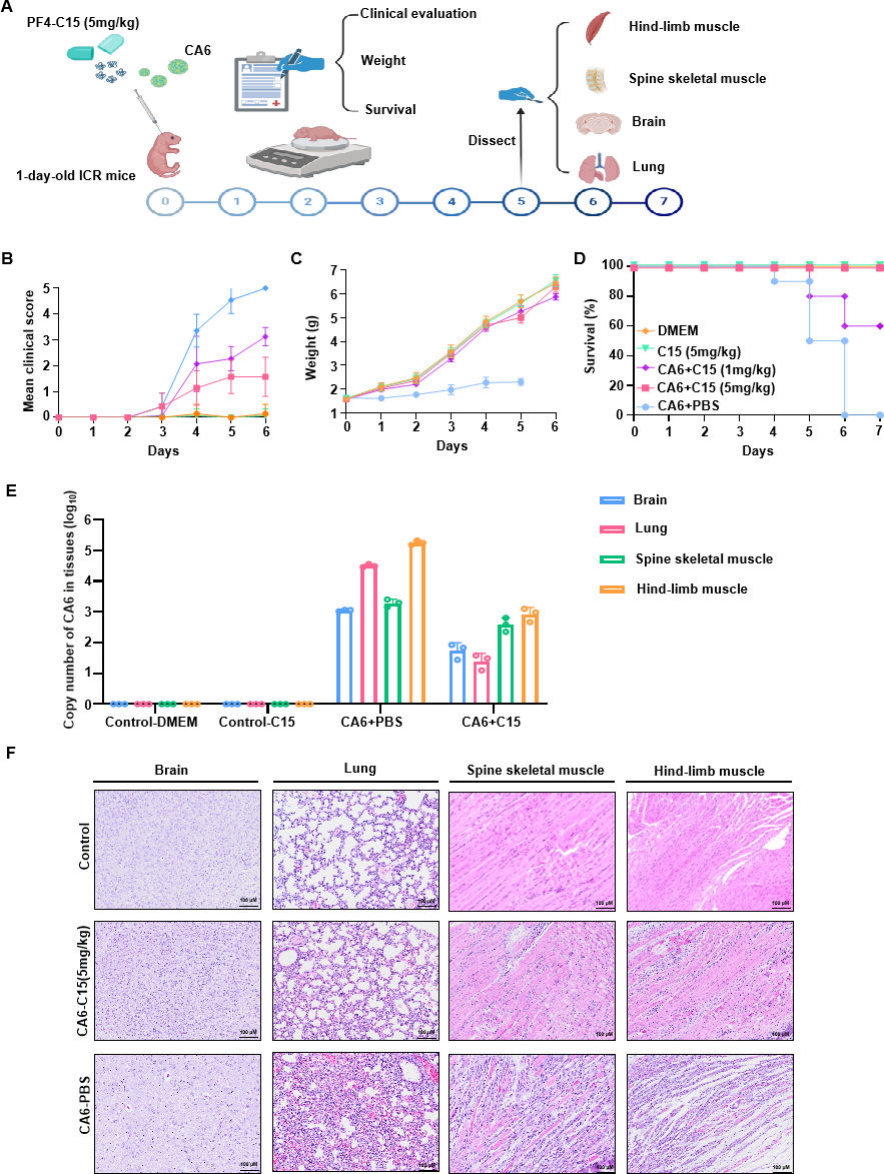
PF4 protects neonatal mice from CA6 lethal challenge. (**A**) The CA6 virus (10^7^ TCID_50_/mL) with varying concentrations of C15 (1 and 5 mg/kg) was intracranially injected into 1 day-old ICR mice (10 µL/mouse). Various indicators were observed over 7 days, and samples were collected for analysis. (**B**) Clinical scores and survival rates were monitored for 6 days post-infection. Clinical disease severity was categorized as follows: 0, healthy; 1, lethargy and inactivity; 2, wasting; 3, limb tremor and weakness; 4, hind-limb paralysis; and 5, moribund or dead. (**C**) Changes in body weight of the mice over a 7 day period were recorded. (**D**) Survival rates of the mice over 7 days were evaluated. (**E**) Copy numbers of CA6 in brain, lung, spinal skeletal muscle, and hind-limb muscle tissues of infected mice were assessed by RT-qPCR on day 4. (**F**) Representative images of hematoxylin and eosin-stained tissues from mice subjected to various treatments were captured on day 4. Magnification: ×200. Scale bars: 100 µm.

To investigate the anti-viral efficacy of C15 against CA6 *in vivo*, we established a lethal infection model using 1-day-old ICR mice. The mice were infected with CA6 at a concentration of 10^7^ TCID_50_ and then treated with two different concentrations of C15 (1 and 5 mg/kg). The mice were monitored over a period of 7 days to assess various indicators (Fig. 7A). The mice in the control group, which received Dulbecco’s Modified Eagle Medium (DMEM) alone, began to succumb to the infection on day 4, with all mice dying by day 6. On day 6, the average clinical score of the mice in the control group was 5. In contrast, both concentrations of C15 significantly alleviated the clinical symptoms associated with infection compared to the control treatment (Fig. 7B). C15 also inhibited infection-induced weight loss. At the lower concentration (1 mg/kg), there was insignificant weight loss, whereas at the higher concentration (5 mg/kg), weight gain approached normal levels (Fig. 7C). Treatment with 1 mg/kg C15 improved survival rates by 60%, while 5 mg/kg C15 provided complete protection, achieving a 100% survival rate (Fig. 7D). Additionally, mice treated with 5 mg/kg C15 without CA6 infection did not exhibit any significant differences in clinical scores, body weight, or survival rates compared to the DMEM control group, indicating that C15 has no toxicity in neonatal mice.

To further confirm the anti-CA6 effects of C15, we measured viral mRNA levels in the brain, lungs, spinal muscles, and hind-limb muscle tissues of CA6-infected mice. C15 treatment decreased VP1 mRNA levels in the brain, lungs, spinal muscles, and hind-limb muscles compared to the control group (Fig. 7E). Histological examination of the lung, spinal skeletal muscle, hind-limb muscle, and brain tissues using hematoxylin and eosin (H&E) staining (Fig. 7F) revealed significant solidification of lung tissue, alveolar rupture, dissolution, rupture of muscle fibers and vacuoles appeared in the brain tissue in the CA6-infected group. In the C15 treatment group, these lung and brain lesions and muscle tissue ruptures were markedly milder than those in the CA6-infected group. Taken together, these results indicate that C15 reduces tissue damage caused by CA6 infection and improves the survival rate of CA6-infected mice.

## DISCUSSION

EV71, CA16, and CA6 are types of EV-A that primarily cause HFMD in children under 5 years old. HFMD epidemics have been increasingly reported across the Asia-Pacific region, including China, Japan, Taiwan, Hong Kong, Malaysia, Singapore, Vietnam, Thailand, and Cambodia (2). In China alone, HFMD cases surged from 1.3 million cases in 2009 to 2.7 million in 2014, with over 2.0 million cases reported annually until the coronavirus disease 2019 outbreak in 2019. After the pandemic, the number of cases rebounded to over 1.68 million in 2023 (https://m.chinacdc.cn). Surveillance studies have shown that EV71 and CA16 often co-circulate, while CA6 and CA10 also co-circulate, leading to intertypic recombination and the emergence of new EV variants (2, 36, 37). Echoviruses and coxsackieviruses (CVB1-6), belonging to the EV-B type, are associated with severe diseases, including AFP, encephalitis, meningitis, and myocarditis (38, 39). Recent AFP surveillance in China, Spain, and West Africa reported that 100%, 81%, and 90% of positive EV samples from patients with AFP, respectively, belong to the EV-B type (40–42). The re-emerging pathogen in 2014, EVD68, classified under the EV-D type, is also linked to AFP as EVD68 exhibits high homology to several EVs, which are known to cause severe diseases (43). Some EV types follow a biennial pattern, co-circulating, or alternative epidemic. However, no multivalent vaccines are currently available to prevent these unpredicted EV epidemics, underscoring the urgent need for broad-spectrum anti-viral drugs to combat EV-associated diseases.

The structural proteins VP1, VP2, VP3, and VP4 form the EV capsids. VP1, VP2, and VP3 are found on the external surface of the capsid, while VP4 is inside. VP1 and VP3 form canyons essential for receptor binding, enabling viral attachment and triggering uncoating (31). The highly conserved hydrophobic pocket within VP1 is a most common target for developing broad-spectrum anti-viral drugs against multiple EV serotypes (44). In this study, we identified peptide C15, derived from PF4, as a broad-spectrum anti-viral agent that specifically targets the VP3 proteins of EVs rather than VP1. Moreover, the VP3 sequences of the different EVs share approximately 70% homology, and we identified the conserved domain 155–170 within VP3 as essential for C15 interaction (Fig. 5 and 6). This domain carries a net-negative charge due to the presence of D at position 156，and this site are conserved in EVs. EVs are known to have negatively charged surfaces (33, 34). In contrast, C15 has a net-positive charge because of its four K residues. We demonstrated a direct charged-based interaction between the negatively charged D at position 156 in VP3 and the positively charged K in C15, which is necessary for PF4’s inhibition of EV replication.

PF4 is a chemokine abundantly secreted by activated platelets and plays a complex role in various viral infections. It has been described as a broad-spectrum inhibitor of HIV-1, influenza virus strain H1N1, and RSV infections (16, 22, 29). In this study, we demonstrated that C15 inhibits the host cell attachment and entry of multiple EVs, including EV71, CA16, CA6, and EVD68. Therefore, it remains to be investigated whether C15 also inhibits HIV-1, H1N1, and RSV. In addition, HIV-1 inhibition by PF4 occurs during virus attachment and entry by directly binding to the viral envelope glycoprotein gp120 and the HIV receptor CXCR4 (22). PF4 also inhibits RSV replication by blocking viral attachment to heparan sulfate (HS) on the surface of host cells (29). Our previous study demonstrated that PF4 simultaneously interacts with VP3 of EV71 and CA16, as well as SCARB2, thereby interfering with the interaction between these viruses and their receptors (27). This study showed that peptide C15 binds specifically to VP3 of EVs. Given that EVs utilize different receptors to attach to and enter host cells, further investigation is needed to determine whether C15 interacts with a common receptor or multiple receptors to disrupt these virus-receptor interactions.

**Fig 8:**
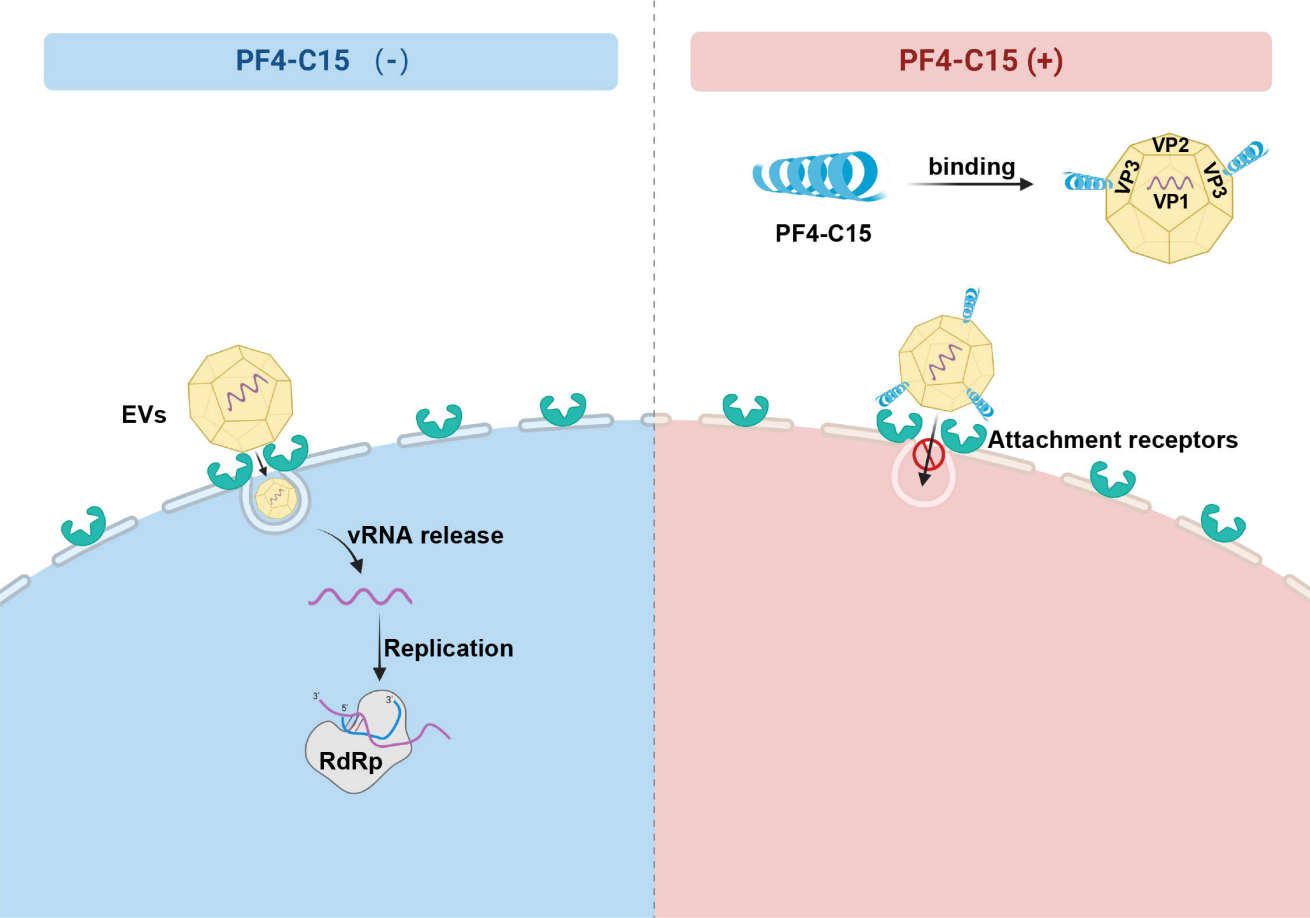
Inhibition mechanism of C15 on enteroviruses. C15 with positive charge binds to the VP3 proteins of EVs, which possess the conserved domain 155–170 with negative charge via a direct ionic interaction, thereby blocking the attachment and entry of EVs into cells.

As a well-studied representative strain of the EV family, EV71 has been used extensively in lethal infection models (9). In this study, we established a neonatal mouse model of lethal CA6 infection. Preincubation of C15 with CA6 protected mice from a CA6 lethal challenge, significantly improving their survival rate (Fig. 7). Previous reports suggest that PF4 induction may be associated with vaccine-induced thrombotic thrombocytopenia (VITT) (45). However, in our experiments, the injection of C15 alone in mice did not result in VITT-related symptoms, such as weight loss, nosebleeds, gum bleeding, or gastrointestinal bleeding. Therefore, C15 can be considered safe at certain concentrations. Nonetheless, our research has limitations, and the data obtained from cell and animal studies may not fully represent the effects that C15 could have in humans. In summary, PF4 blocks the attachment and entry of multiple EVs by binding to VP3 proteins, thereby preventing the interaction between virions and receptors (Fig. 8). This study suggests that the PF4-derived broad-spectrum small peptide C15 is a promising candidate for inhibiting EVs.

## MATERIALS AND METHODS

### Cells and viruses

HEK293T cells (ATCC, #CRL-11268) and human RD cells (ATCC, #CCL-136) were bought from ATCC. The cell culture plates were incubated in a humidified incubator containing 5% CO_2_ at 37°C. All cell lines were tested for *Mycoplasma* contamination. The source of viruses had been reported previously and is as follows: EVD68 (US/KY/14–18953, GenBank: KM851231.1); CA6 (changchun046/CHN/2013, GenBank: KT779410.1); EV71 (changchun063/CHN/2013); CA16 (changchun024/CHN/2013, GenBank: KF055238.1) (46, 47); and CVB3 (Nancy, GenBank: JX312064.1). Echo7 is a gift from Prof. H.L. Wang (48). All viruses were amplified in RD cells.

### Plasmid construction and peptide synthesis

The cDNA of the PF4-Myc with an Myc tag at the N terminals was synthesized and inserted into VR1012 by Comate Bioscience Company (Changchun, China). VR1012 is an efficient eukaryotic expression vector encoding kanamycin resistance, with a cytomegalovirus (CMV) promoter and a bGH polyA signal. The cDNA of CA6/EVD68-VP3 with an HA tag at the N terminals was synthesized and inserted into VR1012 by Comate Bioscience Company. The cDNA of CA6/EVD68-VP1 and VP2 was constructed previously (49). The cDNA of the GFP-C15 (C15 located at the C terminals of GFP) was synthesized and inserted into p-EGFP by Comate Bioscience Company. The mutant plasmids were constructed based on the above plasmids. C15, C15 K-A, and C15 K-M were synthesized by Sangon Biotec Company (Changchun, CHN). The purity of the peptides has reached more than 99%.

### Transfection and infection

cDNA transfections were carried out by Lipofectamine 3000 Reagent (Invitrogen, #L3000-008) according to the manufacturer’s instructions. In the infection experiment, 12 h prior to infection, cells were seeded in a 12-well plate to reach approximately 70% confluence. The virus was mixed in DMEM containing 2% fetal bovine serum (FBS) according to the calculated MOI. This virus mixture was added to the wells of the 12-well plate for infection. During the infection period, the plate was gently shaken several times to ensure uniform distribution of the virus. After 4 h of incubation, the cells were washed twice with phosphate-buffered saline (PBS). The medium was then replaced with DMEM containing 10% FBS. Cells and supernatant were collected 48 h post-infection for subsequent analysis.

### Protein purification

The cDNA of PF4-His with His tag at the N-terminus was synthesized and inserted into PEXs-DH by Comate Bioscience Company and expressed in *E. coli* BL21-derived cells. The proteins were purified by Ni-column affinity chromatography, and a gel filtration column connected to an ÄKTA system (General Electric Company, Connecticut, USA). The purity of the resulting proteins was determined through BCA Protein Assay Kits (Epizyme, #ZJ102L) analysis.

### Peptide-drug configuration and administration

In the *in vitro* experiment, the peptide drug was dissolved in PBS, and the drug was added to the cell culture medium according to the proportion. In the *in vivo* experiment, the drug was dissolved in PBS, added to the virus suspension in the calculated proportion, and injected into mice intracranially.

### RNA extraction and RT-qPCR

RNA from tissues or cells was extracted using Trizol reagent. Twenty percent chloroform was added, mixed thoroughly, and then centrifuged at 4°C. The upper solution was carefully collected, and an equal volume of isopropanol was added. The solution was then incubated at −20°C overnight. On the second day, RNA was precipitated by centrifugation, washed with ethanol, and dissolved in water treated with diethyl pyrocarbonate for quantification. cDNA synthesis was performed using a cDNA Reverse Transcription Kit (TransGen, #AE311-02, Beijing, China) with random or oligo(dT) primers according to the manufacturer’s instructions. The reverse transcription reaction was set up in a 20 µL volume with 2 µg of RNA. For the RT-qPCR assay, a 20 µL reaction mix was prepared, including 0.1 µL (50 µM/L) of each oligonucleotide primer, 2 µg of cDNA template, and 10 µL of 2× SYBR Green Mix (Roche, #491314001, Switzerland) containing HotMaster Taq DNA Polymerase. RT-qPCR was performed using a Roche Z480 instrument with the following cycling conditions: 35 cycles of 95°C for 15 s, 58°C for 15 s, and 68°C for 20 s.

### Western blot and antibodies

After the cell samples were lysed with 1× loading buffer (0.08 M Tris, pH 6.8, with 2.0% SDS, 10% glycerol, 0.1 M dithiothreitol, and 0.2% bromophenol blue), the samples were boiled at 100°C for 15 min and then centrifuged at 12,000 × *g* for 10 min. The proteins were resolved by SDS-PAGE (12.5% or 15%) and transferred to polyvinylidene difluoride membranes for Western blot. After blocking with 3% bovine serum albumin-Tris buffered saline (BSA-TBS) for 1 h at room temperature (RT), the membranes were incubated with primary antibodies at 4°C overnight, followed by corresponding horseradish peroxidase-conjugated secondary antibody (Jackson ImmunoResearch, West Grove, PA, USA) for 1 h at RT. Proteins were developed using an ultrasensitive ECL chemiluminescence detection kit (Epizyme, SQ201).

The following antibodies were used in this study: EV71-VP1 (GeneTex, catalog #GTX132339); CA6-VP1 (GeneTex, catalog #GTX132346); and EVD68-VP1 (GeneTex, catalog #GTX132313); antibodies to CA16-VP1 are laboratory-made, and the specific methods were described previously (50); PF4 antibody (Proteintech, #21157–1-AP); anti-HA polyclonal Ab (Invitrogen, catalog #71–5500); and anti-Myc (Proteintech, #16286–1-AP).

### Co-IP assay

HEK293T cells were transfected with cDNAs for 48 h as indicated. The cells were then harvested and washed with cold PBS followed by disruption with lysis buffer (50 mM Tris-HCl, pH 7.5, 150 mM NaCl, 0.5% NP40, TritonX-100 [1:1,000], and complete protease inhibitor cocktail [Roche, #11836170001]) at 4°C for 4 h. Cell lysates were clarified by centrifugation at 12,000 × *g* for 10 min at 4°C. The corresponding antibody and protein G agarose beads (Roche, #11243233001) were mixed with the precleared cell lysates and incubated at 4°C for 4 h on an endover-end rocker. The reaction mixtures were then washed eight times with cold wash buffer (20 mM Tris-HCl, pH 7.5, 100 mM NaCl, 0.1 mM EDTA, and 0.05% Tween 20) and subsequently analyzed by Western blot.

### Cytotoxicity assays

The cytotoxicity assays of C15 against RD cells were determined using a cell viability assay in 24-well plates with cells cultured to 70%–80% confluence. The cells were treated with various concentrations of C15 dissolved in PBS for 48 h, and then cell viability was assayed using a Cell Counting Kit-8 (CCK-8; Beyotime, #C0037, Shanghai, China). In brief, 10% of the CCK-8 solution was added, and the cells were incubated for about 1 h. The medium without cells was used as a blank control. Absorbance at a wavelength of 450 nm was measured using a microplate reader (CLARIOstar; BMG LABtech, Germany). Cell viability was determined as a percentage of that of the control. All cell proliferation assays were performed in triplicate and repeated in three independent experiments.

### Peptide-virus binding assay

Peptides (1.0 µg per well) dissolved in H_₂_O were coated onto ELISA plates and incubated at 4°C for 2 h. The plates were then blocked with 2% BSA at 4°C overnight. For virus binding, viruses were diluted in PBS and then were added to an ELISA plate to interact with the coated peptides at RT for 1 h. After incubation, unbound viruses were removed by washing with cold PBS. The bound viruses were then lysed for subsequent RT-qPCR analysis.

### Viral adhesion assays

RD cells in six-well plates were infected with CA6 (MOI = 0.5) and EVD68 viruses with C15 bound (100 and 1,000 nM) on shaker at 4°C for 1 h for virus adhesion. The cells were then washed three times with cold PBS and lysed, and vRNA levels were measured by RT-qPCR. For fluorescence experiments, HeLa cells were fixed with 4% paraformaldehyde at 37°C for 15 min following the PBS wash from the previous step. Cells were then permeabilized with saponin (Beyotime, #P0095), washed three times with PBS, and incubated with EVD68 antibody at RT for 1 h. After three PBS washes, the cells were incubated with an Alexa Fluor−488 (Thermo, A32731)-conjugated secondary antibody for 1 h, followed by three additional PBS washes. Finally, a mounting medium containing DAPI was added. Nikon laser confocal microscope (AXR Ti2-E) instrument was used for three-dimensional imaging.

### Neonatal mouse infection model

ICR pregnant specific pathogen-free mice were purchased from Beijing Vital River Laboratory. Pregnant mice gave birth approximately 5 days after arrival at the laboratory. One day-old ICR neonatal mice were used to establish the CA6 infection mouse model. The CA6 virus, at a concentration of 10^7^ TCID_50_/mL, was mixed with varying concentrations of C15 (1 and 5 mg/kg) and intracranially injected into the 1 day-old neonatal mice (10 µL per mouse). Five experimental groups were designed, and observations of various indicators were conducted over the subsequent 7 days, as well as samples collected for analysis. The control group received an intracranial injection of DMEM.

### H&E

All dissected mice were anesthetized prior to organ collection. The brain, lung, spinal skeletal muscle, and hind-limb muscle tissues were harvested. The organs were fixed in 4% paraformaldehyde solution for 2 days. Following fixation, the tissues were dehydrated through an ethanol gradient, cleared with dimethylbenzene, and embedded in paraffin. Sections of 4 µm thickness were prepared for H&E staining. Histopathological analysis of the tissues was conducted using a light microscope, and images were captured with the Olympus VS200 whole-slide scanning system.

### Statistical analysis

Data were obtained from more than three independent experiments. Statistical significance was assessed by two-sided unpaired *t*-tests and one-way analysis of variance compared with the control group (no significance; *P < 0.05, **P < 0.01, ***P < 0.001).

## Data Availability

All data related to this research are listed in the main text or supplemental material and are available from the corresponding author (W.Z.) upon reasonable request.
